# Drug Cocktail Optimization in Chemotherapy of Cancer

**DOI:** 10.1371/journal.pone.0051020

**Published:** 2012-12-07

**Authors:** Saskia Preissner, Mathias Dunkel, Michael F. Hoffmann, Sarah C. Preissner, Nikolai Genov, Wen Wei Rong, Robert Preissner, Karlheinz Seeger

**Affiliations:** 1 Charité - Universitätsmedizin Berlin, Dental, Oral and Maxillary Medicine, CC3, Department of Operative and Preventive Dentistry, Berlin, Germany; 2 Charité – Universitätsmedizin Berlin, Institute for Physiology, CC2, Structural Bioinformatics Group, Berlin, Germany; 3 Charité – Universitätsmedizin Berlin, Pediatric and Adolescent Medicine, CC17, Department of Pediatrics, Division of Oncology and Hematology, Berlin, Germany; Southern Illinois University School of Medicine, United States of America

## Abstract

**Background:**

In general, drug metabolism has to be considered to avoid adverse effects and ineffective therapy. In particular, chemotherapeutic drug cocktails strain drug metabolizing enzymes especially the cytochrome P450 family (CYP). Furthermore, a number of important chemotherapeutic drugs such as cyclophosphamide, ifosfamide, tamoxifen or procarbazine are administered as prodrugs and have to be activated by CYP. Therefore, the genetic variability of these enzymes should be taken into account to design appropriate therapeutic regimens to avoid inadequate drug administration, toxicity and inefficiency.

**Objective:**

The aim of this work was to find drug interactions and to avoid side effects or ineffective therapy in chemotherapy.

**Data sources and methods:**

Information on drug administration in the therapy of leukemia and their drug metabolism was collected from scientific literature and various web resources. We carried out an automated textmining approach. Abstracts of PubMed were filtered for relevant articles using specific keywords. Abstracts were automatically screened for antineoplastic drugs and their synonyms in combination with a set of human CYPs in title or abstract.

**Results:**

We present a comprehensive analysis of over 100 common cancer treatment regimens regarding drug-drug interactions and present alternatives avoiding CYP overload. Typical concomitant medication, e.g. antiemetics or antibiotics is a preferred subject to improvement. A webtool, which allows drug cocktail optimization was developed and is publicly available on http://bioinformatics.charite.de/chemotherapy.

## Introduction

### Drug metabolism and drug-drug interactions

Drug metabolism is a complex biochemical network, which consists of many different reactions and pathways in the human organism. Some drugs are excreted unchanged in urine and faeces without metabolic degradation in the liver, but most drugs undergo a multi-step metabolism, which is mainly accomplished by enzymes of the cytochrome P450 family (CYP). CYP catalyze a large amount of enzymatic reactions, such as alcohol oxidations, dehydrogenation and isomerizations. It is a difficult task of medical science and daily clinical practice to establish effective combinations of drugs that do not affect each other's metabolic pathways.

The Human Genome Project revealed 57 different CYP variants [1]. The variant biological activities and specificity among each single CYP are an important issue for researchers as well as physicians. The knowledge of level and catalytic activity of the specific CYP and the effect on drug metabolism could and should lead to personalized drug dosages to optimize the therapeutic effect and minimize harmful side effects. Furthermore, the induction of a CYP by a drug, which is also active in another drug's metabolism, requires increase of the dosage of the first drug to achieve the same therapeutic effect. In case of inhibition, the dosage should be reduced, resulting in diminished side effects. In addition, the drug excretion pathway through kidney has also an important influence on individual drug response. Unfortunately, drugs that are mainly removed by this pathway from the body, will accumulate if an impaired kidney function exists. Therefore, the extrarenal fraction (**Q_0_**) value is able to predict whether a drug is primarily excreted unchanged via kidneys or metabolized and/or removed through another pathway. Thereby is (1- **Q_0_**) the fraction, which is removed unchanged via kidneys. High **Q_0_** values stand for mainly metabolized drugs and/or kidney independent excretion. In order to prevent adverse side effects and toxic drug levels in diseased kidney patients the **Q_0_** value should be taken into account to change the drug or adjust the dosage.

Due to multi-drug administration in polychemotherapeutic regimens, adverse side effects are discussed intensely in pharmaceutical research [2]. Three frequently occurring problems should be considered:

1. Adverse side effects because of limited capacity of metabolizing enzymes,
2. Malfunctioning in-vivo activation of prodrugs due to inhibited or mutated CYPs,
3. Unexpected drug levels because of enzyme induction or inhibition.

### CYPs in chemotherapy

In this manuscript, we focus on leukemia while other types of cancer (soft-tissue sarcoma, osteosarcoma, nephroblastoma, neuroblastoma, brain tumors, hodgkin-lymphoma, non-hodgkin lymphoma, low-grade glyoma, and germ cell tumors) are considered at the website.

Most subtypes of leukemia are primarily treated with risk-adapted polychemotherapy protocols, which consist of induction, consolidation, re-induction and maintenance regimens. For risk-adaptation certain prognostic factors are applied, such as leukocyte cell count, age, gender, cytogenetic findings and response to induction therapy [3]. Patients receive up to 13 different antineoplastic drugs. In leukemia, disease progression can be influenced by genetic variants encoding proteases, angiogenic factors, hematopoietic cytokines, bone marrow stroma factors or structural proteins in epithelium. Due to scientific progress individualized medicine is being increasingly developed in the last years and CYP-drug, as well as drug-drug interactions are being considered [4], [5]. Individualized medicine also deals with single nucleotide polymorphisms (SNPs) of CYPs to predict patient responses [6], [7].

In children with acute lymphoblastic leukemia (ALL) an increased risk of vincristine polyneurotoxicity associated with low CYP 3A5 expression has been reported [8].

Many antineoplastic agents are prodrugs, e.g. cyclophosphamide, ifosfamide, dacarbazine, procarbazine and tamoxifen, requiring in vivo activation by CYPs [9]. An inhibition of CYPs due to multidrug administration could potentially affect negatively the therapeutic efficacy. The clinical relevance of such considerations was shown in several clinical trials, where CYPs and SNPs play a role in potentially preventing treatment related deaths [9], [10], [11], [12], [13]. A retrospective study showed a 3-fold higher risk of death in patients with a polymorphism of CYP3A4 who were receiving cyclophosphamide-based adjuvant chemotherapy [14].

These findings suggest that individual SNPs in CYPs and drug-drug interactions in polychemotherapy are important issues and treatment regimens should be reevaluated regarding such interactions.

## Materials and Methods

### Treatment regimens

Information on drug administration of chemotherapeuticals in oncology and their drug metabolism was collected from scientific literature and various web resources. About 100 common treatment regimens were extracted from the blue book [15].

### CYP-drug interactions

The drugs from the treatment regimens were subdivided into two groups regarding their purpose:

- Antineoplastic agents
- Supportive treatment, e.g. antiemetics, antimycotics, antibiotics

Information on CYP metabolism was also extracted from Nelsons Homepage [16], Flockharts Interaction Table [17], University of Maryland's Drug Checker, PubChem [18], PDB [19]. Some information was gathered from FDA-files.

### Textmining

The flood of information on drugs in the world wide web (WWW) is overwhelming [20]. The World Wide Web Cosortium aims at converting the existing web into a Semantic Web or “web of data” [21]. Accordingly, we carried out a new textmining approach using Semantic Web Standards. For the development of the CYP-specialized textmining pipeline we used the literature and information retrieval packages Lucene and LingPipe. Therefore, the complete Medline/PubMed data was downloaded from the NCBI FTP site in xml-format and indexed. The indexed data is dynamically queried by a search engine written in Java resulting in a sql-file containing the textmining hits. The search engine comprises several lists of synonyms for identifying entities like chemical compounds, biological targets, genes, cell-types, polymorphisms as well as interaction related entities. Abstracts were automatically screened for antineoplastic drugs and their synonyms in combination with a set of human CYPs in title or abstract. Furthermore, the relation between drug and CYP was classified according to interaction terms like “inhibit”, “induce”, “metabolize” etc. The query was: (DrugSynonym[ti] AND CypSynonym[ti]) OR (DrugSynonym[abstract] AND InteractionTerm AND CypSynonym[abstract]). There was a need for restricting positional distance between occurrences of the terms, e.g. if terms are found far from each other in a paper. Those 2,060 records found were scored rule-based to identify relations between entities. The rules employ order, redundancy and distance between entities, topic segmentation and sentence breaking for boundaries. Duplicates were removed and a team of scientists manually processed 723 papers found in PubMed. Each drug was attributed to those CYPs that are involved in drug metabolism as substrate, inhibitor or inducer.

### ATC Classification System

Many problems, such as enzyme overload, enzyme induction or inhibition occur in combination therapy of leukemia. Some of these drug-drug interactions can be avoided by choosing an alternative drug. Based on the WHO classification system, that classifies drugs into different groups according to *A*natomic site of action, *T*herapeutical effect and *C*hemical structure (ATC), alternative drugs could be administered. Additionally, the suggestions of alternative drugs were manually curated by oncologists and checked for sanity.

### Database

To overcome these problems, we generated a web-interface for clinicians to check drug-drug interactions. The database provides information on drug metabolism including PubMed references. The database is designed as a relational database on a MySQL server. For chemical functionality, the MyChem package is included, which aims to provide a complete set of functions for handling chemical data within MySQL. The website is built with PHP and javascript, web access is enabled via Apache Webserver 2.2.

## Results and Discussion

Those 2,060 records were found through the automated textmining approach. Another 50 records were manually identified. 864 duplicates were automatically removed and another 92 records were excluded. A team of scientists manually processed 723 papers found in PubMed. There are a lot of undesired drug-drug interactions via CYPs. In particular, the number and effect of anti-neoplastic drugs often cause severe problems, possibly ending up with death. The extensive search revealed three CYPs, which are mainly involved in the metabolism of antineoplastic agents.

**Figure 1:**
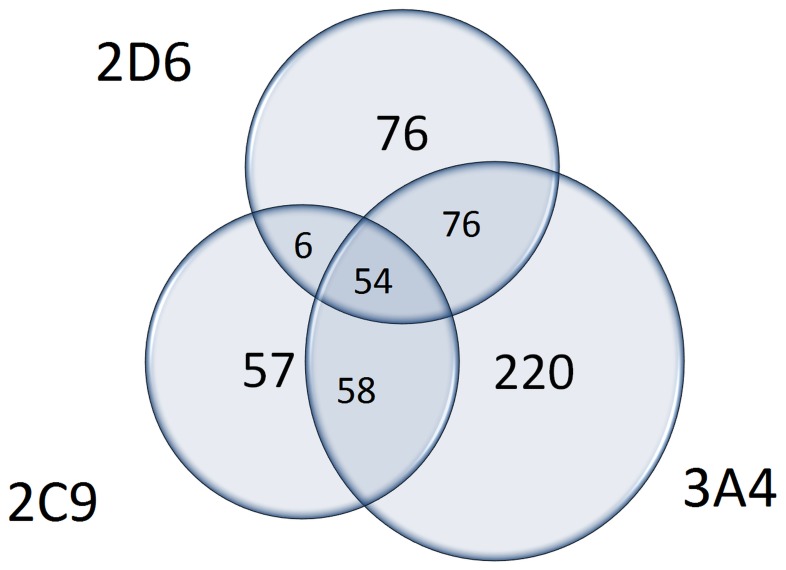
The Venn diagram illustrates the enzyme overload of CYPs 3A4, 2C9, 2D6 in chemotherapy. The numbers within the circles represent the drugs, which are metabolized by the CYPs. Intersection areas show the drugs, which are metabolized by two or three of the CYPs.

Figure 1 shows these CYPs, namely CYP 3A4, 2D6 and 2C9, which are involved in the metabolism of most of the drugs. Interestingly, CYPs 2D6 and 2C9 are highly polymorphic, which makes it even more important to disencumber the CYPs from some drugs and in second step, trying to use different metabolic pathways.

We have analyzed the antineoplastic drugs from over 100 treatment regimens regarding their drug metabolism. The results are summarized in Table S1 of Supporting Information.

To optimize therapeutic regimens, the effect of supportive drugs like antibiotics, antimycotics, antiemetics etc. in the metabolic process have to be taken into account, which are shown in Table S2 of Supporting Information.

**Figure 2:**
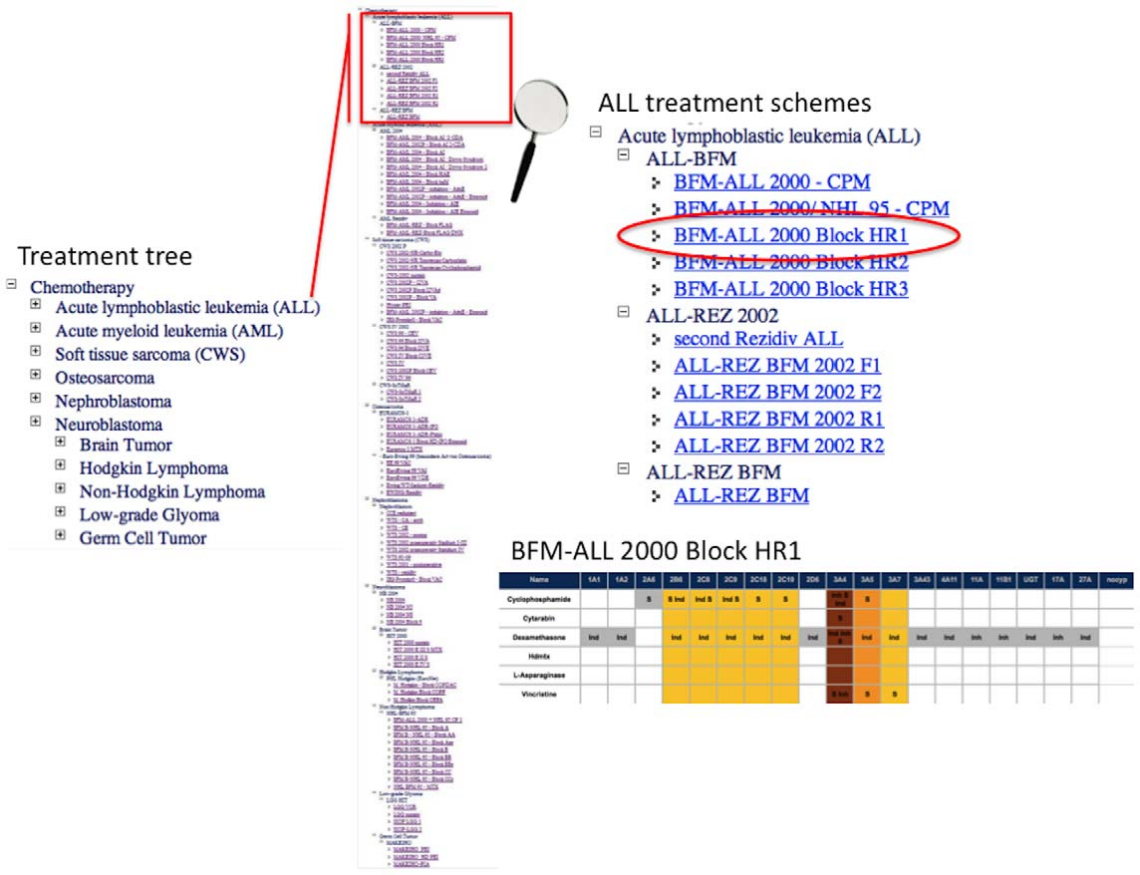
Treatment algorithm: Different antineoplastic treatment regimens in chemotherapy, ordered by diseases. By clicking on one of the diseases, different treatment options open up. After choosing one treatment regimen the metabolism of that drug-cocktail is illustrated.

These analyses suggest several drug-drug interactions, but also show some alternatives to avoid enzyme overload or induction. Additionally, the analysis of the ATC codes for drug classification and the addressed targets provide hints for possible alternative medication. Going through the list of chemotherapeutic drugs and supportive medication, we have compiled a comprehensive list of combination therapies, which are optimized regarding their metabolism. This list is structured according to an algorithm starting from the different cancer types, different therapy cycles, relapse etc. (Figure 2).

Furthermore, the **Q_0_** and elimination half-life (EHL) values are displayed to compare the pharmacological properties of drugs and their alternatives. On the one hand, longer EHL potentially means CYP overload and should be avoided, on the other hand the effective presence of the drugs has to be longer than the cell cycle of the cancer cells (re-dosing may be required for shorter EHLs). The consideration of individual pharmacokinetic parameters like Km and Vmax for drugs and CYPs [22] would be desirable but requires refined models for each particular drug-drug interaction (reversible, competitive, non-competitive, uncompetitive, irreversible etc.), which remains a future goal. Beside the role in patients with nephropathies, the **Q_0_** could also help to estimate the extent of CYP-drug interactions. Drugs with low **Q_0_** values (<0.3) are excreted unchanged to a large extent and occupying the CYP system lesser. In conclusion, their impact on interactions is lower than for drugs with higher **Q_0_** values. Hence, consideration of **Q_0_** values in finding alternative drugs is useful to reduce the interaction potential, if the function of kidneys is sufficient. However, limitations are a small number of eligible drugs with low **Q_0_** values, and that high values do not necessarily mean more CYP reactions. But it provides a useful support to select the alternative drugs.

To exemplify here, we chose one typical treatment protocol for the treatment of ALL, which consists of the antineoplastic drugs cyclophosphamide, cytarabine, HDMTX, L-asparaginase and vincristine, as well as the corticosteroids prednisone/dexamethasone.

**Figure 3:**
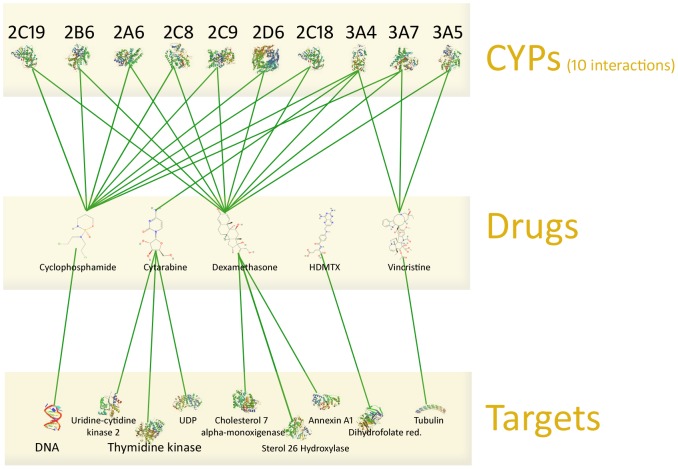
CYP interactions and targets of treatment regimen before optimization. The drugs of the medication are listed centrally in the Figure. Several green lines heading upwards illustrate ten CYPs, which are involved in the metabolism. The green lines heading downwards show the targets, which are metabolized by these drugs.

CYPs involved in the metabolism of the mentioned drugs are listed in Table 1, ordered by substrate, inducer and inhibitor. It is clearly visible, that many CYPs are involved in the metabolism processes, ending up in eleven interactions. These are illustrated with targets in Figure 3.

**Table 1:** Treatment regimen before optimization: Drugs for the treatment of ALL at initial diagnosis.

				Involved CYPs			
Drug	Purpose	Q_0_	EHL	Substrate of	Inducer of	Inhibitor of	References
Cyclophosphamide	Antineoplastic agent	0.75	7	2A6, 2B6, 2C8, 2C9, 2C18, 2C19, 3A4, 3A5	2B6, 2C8, 2C9, 3A4	3A4	[23], [24], [25], [26], [27]
Cytarabine	Antineoplastic agent	0.9	2	3A4			[28]
Dexamethasone	Corticosteroid	0.9	3	3A4	1A1, 1A2, 2B6, 2C8, 2C9, 2C18, 2C19, 2D6, 3A4, 3A5, 3A7, 3A43, 4A11, UGT, 27A	3A4, 11A, 11B1, 17A	[17], [29], [30], [31], [32], [33], [34], [35], [36], [37], [38], [39], [40], [41]
Vincristine	Antineoplastic agent	0.8	85	3A4, 3A5, 3A7		3A4	[17], [42], [43], [44]
HdMTX	Antineoplastic agent						
L-Asparaginase	Antineoplastic agent						

The second, third and fourth columns list the purpose of these drugs, their extrarenal fraction (**Q_0_**) and elimination half-life (EHL), while the next three columns show involved CYPs ordered by substrate, inducer and inhibitor. References are given in the last column.

**Figure 4:**
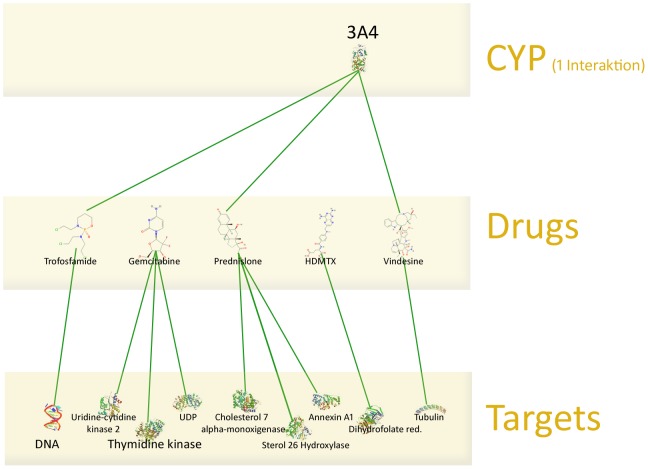
CYP interactions and targets of treatment regimen after optimization. By choosing drugs from the same ATC group with different metabolism pathways, only one CYP interaction remains.

Based on the ATC codes, we extracted an alternative treatment regimen to avoid these interactions. The results are illustrated in Table 2 and Figure 4. Figure 4 shows that there is only one interaction left, while all other interactions could be omitted using different metabolic pathways of other drugs.

**Table 2:** Treatment regimen after optimization: Possible alternatives in the treatment of ALL.

				Involved CYPs			
Drug	Purpose	Q_0_	EHL	Substrate of	Inducer of	Inhibitor of	References
Gemcitabine	Antineoplastic agent	0.9	1.2				
Prednisolone	Corticosteroid	0.7	3	3A4, 3A5	3A4, 3A5	2A6	[45], [46], [47], [48]
Trofosfamide	Antineoplastic agent	0.9	1	2B6, 3A4			[49]
Vindesine	Antineoplastic agent	0.87	24	3A4			[50]
HdMTX	Antineoplastic agent						
L-Asparagine	Antineoplastic agent						

The second, third and fourth columns list the purpose of these drugs, their extrarenal fraction (**Q_0_**) and elimination half-life (EHL) in hours, while the next three columns show involved CYPs ordered by substrate, inducer and inhibitor. References are given in the last column.

### Database

**Figure 5:**
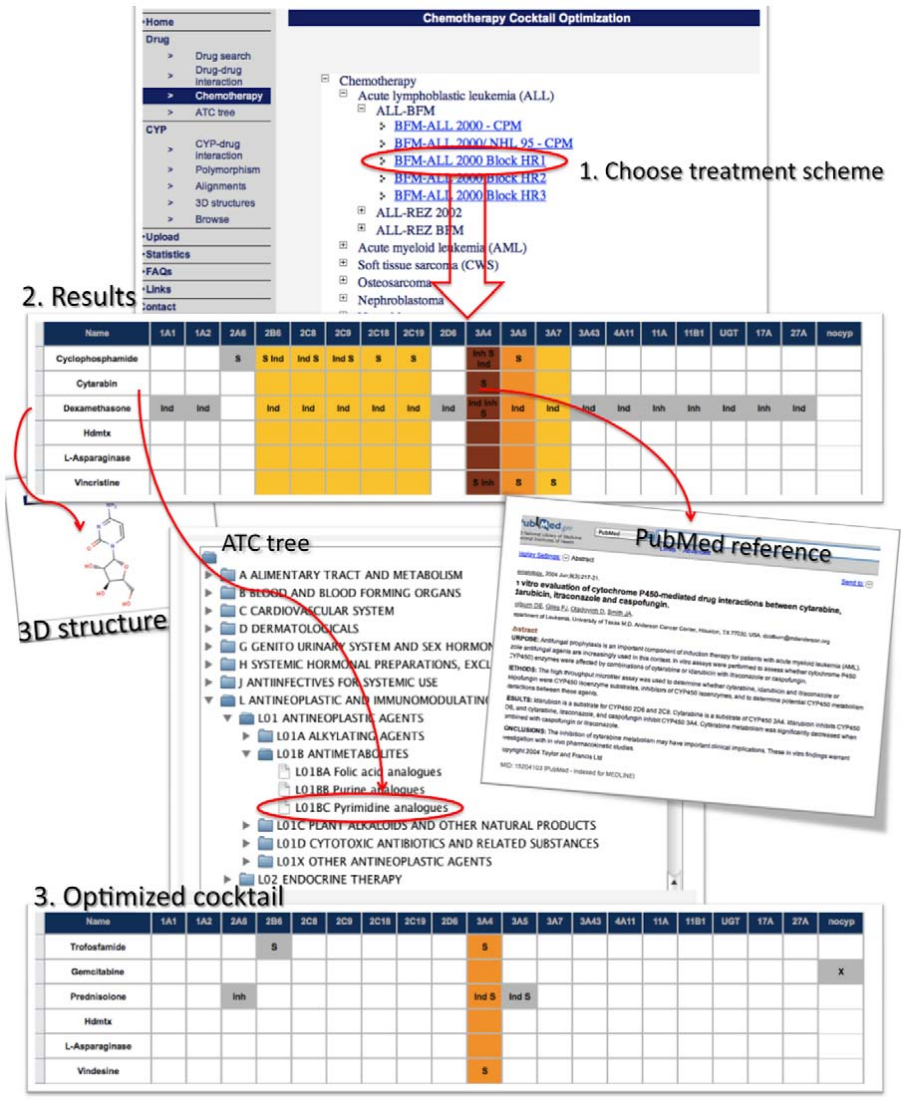
Optimization tool. Clicking on “Chemotherapy” in the navigation directs to the treatment tree, enabling to browse through different treatment regimens ordered by diseases. Once a treatment regimen is chosen, the drug-cocktail is shown on the “Results” page. The enzyme overload is visualized in different colors. PubMed references are indicated, as well as 3D structures of the drugs and the ATC tree defining the purpose of the drugs. Based on the ATC group, several alternatives for each drug are given, providing optimization of the cocktail with less drug-drug-interactions.

We created a web-tool for clinicians to analyze diverse drug-drug interactions of over 100 antineoplastic treatment regimens. Figure 5 shows the main features of the website. To visualize treatment regimens, just click on “Chemotherapy” in the navigation. If your specific drug-cocktail is not in the list, click on “Drug-drug interaction” and type in your medication manually. Once a treatment regimen is chosen or manually typed a drug-cocktail, the database provides a variety of information.

To view drug structures or ATC groups, just click on the drug. CYPs involved in the same metabolic pathway are presented in different columns. “S” means substrate, “E” inducer and “I” inhibitor. Clicking on these abbreviations leads to the PubMed references. Colored columns illustrate the multi-use of specific CYP pathways. Based on ATC-codes, drug alternatives using different metabolic pathways for each drug are presented below, which enables the user to optimize the cocktail regarding its metabolism.

This comprehensive resource is freely available at: http://bioinformatics.charite.de/chemotherapy and is also applicable on smartphones and tablet-PCs.

## Supporting Information

Table S1 **Antineoplastic drugs in polychemotherapy regimens.** Involved CYPs are ordered by substrate “S”, inducer “E” and inhibitor “I”.
(DOCX)

Table S2 **Supportive treatment used in chemotherapy.** Involved CYPs are ordered by substrate “S”, inducer “E” and inhibitor “I”.
(DOCX)
