# Supplementary material for: Drug Cocktail Optimization in Chemotherapy of Cancer
Source: PLoS One. 2012 Dec 7;7(12):e51020. doi: 10.1371/journal.pone.0051020 (PMC3517625; doi:10.1371/journal.pone.0051020)
Supplement: Table S1 — Antineoplastic drugs in polychemotherapy regimens. Involved CYPs are ordered by substrate “S”, inducer “E” and inhibitor “I”. (DOCX) [file pone.0051020.s001.docx]

Table S1:

| Name | 1A1 | 1A2 | 1B1 | 2A6 | 2B6 | 2C8 | 2C9 | 2C18 | 2C19 | 2D6 | 2E1 | 3A4 | 3A5 | 3A7 | UGT | 27A | excretion | nocyp |
| --- | --- | --- | --- | --- | --- | --- | --- | --- | --- | --- | --- | --- | --- | --- | --- | --- | --- | --- |
| Mercaptopurine | S |  |  |  |  |  |  |  |  |  |  |  |  |  | S |  |  | X |
| Thioguanine |  |  |  |  |  |  |  |  |  |  |  |  |  |  |  |  |  | X |
| Bleomycin |  |  |  |  |  |  |  |  |  |  |  |  |  |  |  |  | X |  |
| Carboplatin |  |  |  |  |  |  |  |  |  |  |  | E |  |  |  |  | X |  |
| Carmustine |  | S |  |  |  |  |  |  |  |  |  |  |  |  |  |  |  |  |
| Chlorambucil |  |  |  |  |  |  |  |  |  |  |  |  |  |  |  |  |  | X |
| Cisplatin |  |  |  |  | I |  |  |  |  |  | S E |  |  |  |  |  |  |  |
| Cyclophosphamide |  |  |  | S | S E | E S | E S | S | S |  |  | I S E |  | S |  |  |  |  |
| Ciclosporin |  |  |  |  |  | I | I |  | I | I |  | E S I | I E S | E S |  | I |  |  |
| Cytarabine |  |  |  |  |  |  |  |  |  |  |  | S |  |  |  |  |  |  |
| Dacarbazine | S I | S I |  |  |  |  |  |  |  |  | S |  |  |  |  |  |  |  |
| Daunorubicin | S | S | I |  |  |  |  |  |  |  |  | S I | E |  |  |  |  |  |
| Doxorubicin | E | E | I |  | I |  |  |  |  | I S |  | I E S | S |  |  |  |  |  |
| Epirubicin |  |  |  |  |  |  |  |  |  |  |  |  |  |  | S |  |  |  |
| Etoposide |  | S |  |  |  | I | I |  |  |  | S | S E I | E S |  | S |  |  |  |
| Fludarabine |  |  |  |  |  |  |  |  |  |  |  |  |  |  |  |  | X |  |
| Hydroxycarbamide |  |  |  |  |  |  |  |  |  | I E |  |  |  |  |  |  |  |  |
| Idarubicin |  |  |  |  |  |  | S |  |  | S I |  |  |  |  |  |  |  |  |
| Ifosfamide |  |  |  | S | S | E S | S E | S | S |  |  | I S E | S | S |  |  |  |  |
| imatinib |  | S |  |  |  | S I | S I |  | S | S I |  | S I | S I | S |  |  |  |  |
| Irinotecan |  |  |  |  | S |  |  |  |  |  |  | I S | S | S |  |  |  |  |
| Lomustine |  |  |  |  |  |  |  |  |  | I S |  | I |  |  |  |  |  |  |
| Melphalan |  |  |  |  |  |  |  |  |  |  |  |  |  |  |  |  |  | X |
| Mitoxantrone |  |  | I |  |  |  |  |  |  |  | S | I |  |  |  |  |  |  |
| Pentostatin |  |  |  |  |  |  |  |  |  |  |  |  |  |  |  |  | X |  |
| Procarbazine | S | S | S |  |  |  |  |  |  |  |  |  |  |  |  |  |  |  |
| Temozolomide |  |  |  |  |  |  |  |  |  |  |  | E |  |  |  |  | X |  |
| Topotecan |  |  |  |  |  |  |  |  |  |  |  | E I |  |  |  |  |  |  |
| Trofosfamide |  |  |  |  | S |  |  |  |  |  |  | S |  |  |  |  |  |  |
| Vinblastine |  |  |  |  |  |  |  |  |  | I S |  | S I E | S |  |  |  |  |  |
| Vincristine |  |  |  |  |  |  |  |  |  |  |  | S I | S | S |  |  |  |  |
| Vindesine |  |  |  |  |  |  |  |  |  |  |  | S |  |  |  |  |  |  |
