# Supplementary material for: Drug Cocktail Optimization in Chemotherapy of Cancer
Source: PLoS One. 2012 Dec 7;7(12):e51020. doi: 10.1371/journal.pone.0051020 (PMC3517625; doi:10.1371/journal.pone.0051020)
Supplement: Table S2 — Supportive treatment used in chemotherapy. Involved CYPs are ordered by substrate “S”, inducer “E” and inhibitor “I”. (DOCX) [file pone.0051020.s002.docx]

Table S2:

| Name | 1A1 | 1A2 | 1B1 | 2A6 | 2A13 | 2B6 | 2C8 | 2C9 | 2C18 | 2C19 | 2D6 | 2E1 | 2F1 | 2S1 | 3A4 | 3A5 | 3A7 | 3A43 | 4A11 | 4B1 | 4F2 | 7A1 | 11A | 11B1 | 11B2 | UGT | 17A | 19A | 26A1 | 27A | 51A | excretion | nocyp |
| --- | --- | --- | --- | --- | --- | --- | --- | --- | --- | --- | --- | --- | --- | --- | --- | --- | --- | --- | --- | --- | --- | --- | --- | --- | --- | --- | --- | --- | --- | --- | --- | --- | --- |
| Aciclovir |  |  |  |  |  |  |  |  |  |  |  |  |  |  |  |  |  |  |  |  |  |  |  |  |  |  |  |  |  |  |  |  | X |
| Alizapride |  |  |  |  |  |  |  |  |  |  |  |  |  |  |  |  |  |  |  |  |  |  |  |  |  |  |  |  |  |  |  | X |  |
| Retinol (vit A) | I | S I | S |  |  | I | I S | I |  | I |  | S E |  | S | S |  |  |  |  |  | I | I |  |  |  |  |  |  | E |  |  |  |  |
| Allopurinol |  |  |  |  |  |  |  |  |  |  |  | I |  |  |  |  |  |  |  |  |  |  |  |  |  |  |  |  |  |  |  |  |  |
| Amphotericin B |  |  |  |  |  |  |  |  |  |  |  |  |  |  |  |  |  |  |  |  |  |  |  |  |  |  |  |  |  |  |  | X |  |
| Aprepitant |  | S |  |  |  |  |  | I E |  | I S |  |  |  |  | I E S | S | S |  |  |  |  |  |  |  |  |  |  |  |  |  |  |  |  |
| Carbamazepine |  | S I E |  | E |  | S E | S E | E |  | E I |  |  |  |  | S E | S E | S |  |  |  |  |  |  |  |  | E |  |  |  |  |  |  |  |
| Ciprofloxacin |  | I |  |  |  |  |  |  |  |  |  | E |  |  | I |  |  |  |  |  |  |  |  |  |  |  |  |  |  |  |  |  |  |
| Clarithromycin |  | I |  |  |  |  |  |  |  | S I |  |  |  |  | I E S | S | S |  |  |  |  |  |  |  |  |  |  |  |  |  |  |  |  |
| Clemastine |  |  |  |  |  |  |  |  |  |  | I |  |  |  | I |  |  |  |  |  |  |  |  |  |  |  |  |  |  |  |  |  |  |
| Colistin |  |  |  |  |  |  |  |  |  |  |  |  |  |  |  |  |  |  |  |  |  |  |  |  |  |  |  |  |  |  |  | X |  |
| Cotrimoxazole |  |  |  |  |  |  |  | I S |  |  |  |  |  |  |  |  |  |  |  |  |  |  |  |  |  |  |  |  |  |  |  | X |  |
| Prednisone | E | E |  |  |  |  |  |  |  | E |  |  |  |  | S E I |  |  |  |  |  |  |  |  |  |  |  |  |  |  |  |  |  |  |
| Dexamethasone | E | E |  |  |  | E | E | E | E | E | E |  |  |  | E I S | E S | E S | E | E |  |  |  | I | I |  | E | I |  |  | E |  |  |  |
| Dopamine |  | S I |  |  |  |  |  | S |  | S | S | I |  |  |  |  |  |  |  |  |  |  |  |  |  | S |  | I |  |  |  |  |  |
| Erythromycin | S | I S | S |  |  | S |  |  |  |  |  |  |  |  | I E S | S I | S |  |  |  |  |  |  |  |  |  |  |  |  |  |  |  |  |
| Famotidine |  |  |  |  |  |  |  |  |  | S |  |  |  |  |  |  |  |  |  |  |  |  |  |  |  |  |  |  |  |  |  |  |  |
| Fluconazole | E | I |  |  |  |  | I | I S | I | I | I |  |  |  | I E | I E |  |  |  |  |  |  |  | I |  | S I |  | I |  |  |  |  |  |
| Furosemide |  |  |  |  |  |  |  |  |  |  |  | S |  |  |  |  |  |  |  |  |  |  |  |  |  | S |  |  |  |  |  | X |  |
| Ganciclovir |  |  |  |  |  |  |  |  |  |  |  |  |  |  |  |  |  |  |  |  |  |  |  |  |  |  |  |  |  |  |  | X |  |
| Granisetron | S | S |  |  |  |  |  |  |  |  | I E S |  |  |  | S | S |  |  |  |  |  |  |  |  |  |  |  |  |  |  |  |  |  |
| Itraconazole | E |  |  |  |  |  |  |  |  |  | I | I |  |  | I S |  |  |  |  |  |  |  |  |  |  |  |  | I |  |  |  |  |  |
| Ketoconazole | I E | I | I | I |  | I | I | I |  | I | I | I | I |  | E I S | I E S | I |  |  |  | I |  | I | I |  | S I |  |  | I |  | I | X |  |
| Loperamide |  |  |  |  |  | S | S |  |  |  | S |  |  |  | S I |  |  |  |  |  |  |  |  |  |  |  |  |  |  |  |  |  |  |
| Lynestrenol |  |  |  |  |  |  |  | E |  | S E |  |  |  |  | S |  |  |  |  |  |  |  |  |  |  |  |  |  |  |  |  |  |  |
| Mannitol |  |  |  |  |  |  |  |  |  |  |  |  |  |  |  |  |  |  |  |  |  |  |  |  |  |  |  |  |  |  |  |  | X |
| Mesna |  |  |  |  |  |  |  |  |  |  |  |  |  |  |  |  |  |  |  |  |  |  |  |  |  |  |  |  |  |  |  | X |  |
| Metoclopramide |  |  |  |  |  |  |  |  |  |  | S I |  |  |  |  |  |  |  |  |  |  |  |  | I | E |  | I |  |  |  |  |  |  |
| Omeprazole | E S I | I E S | E |  |  |  | S | I S | S | S E I | I |  |  |  | I S E | I E S |  |  |  |  |  |  | I |  |  | E |  |  |  |  |  |  |  |
| Ondansetron |  | I S |  |  |  |  |  | S |  |  | S I | S |  |  | S E | S | S |  |  |  |  |  |  |  |  |  |  |  |  |  |  |  |  |
| Pantoprazole |  | E S |  |  |  |  |  | I |  | S I |  |  |  |  | S E |  |  |  |  |  |  |  |  |  |  |  |  |  |  |  |  |  |  |
| Paracetamol | S | S |  | S |  |  | S | S |  |  | E S | S E |  |  | S E I | S E |  |  |  |  |  |  |  |  |  | S |  |  |  |  |  |  |  |
| Pethidine |  | E |  |  |  | S |  |  |  | S |  |  |  |  | S E | E |  |  |  |  |  |  |  |  |  |  |  |  |  |  |  |  |  |
| Phenobarbital | E | E | E | I E |  | E | E S | E S | E | S E |  | S E |  |  | E | E | E |  | E | E |  |  |  |  |  | E I |  |  |  |  |  |  |  |
| Phenytoin |  |  |  |  |  | E | E S | S I E | E S | E S |  |  |  |  | E S | E | E |  |  |  |  |  |  | I |  | I S E |  |  |  |  |  |  |  |
| Prednisolone |  |  |  | I |  |  |  |  |  |  |  |  |  |  | E S | S E |  |  |  |  |  |  |  |  |  |  |  |  |  |  |  |  |  |
| Ranitidine |  | I S |  |  |  |  |  |  |  | S | I S |  |  |  | I | I |  |  |  |  |  |  |  |  |  | I |  |  |  |  |  | X |  |
| Rifampicin | E | S E | E | I E S | E | E S | E I S | E S | E | E S | S E | E S | E |  | E I S | E S I | E | E | E |  |  | I |  |  |  | E S |  |  |  |  |  |  |  |
| Sucralfate |  |  |  |  |  |  |  |  |  |  |  |  |  |  |  |  |  |  |  |  |  |  |  |  |  |  |  |  |  |  |  | X |  |
| Trimethoprim |  |  |  |  |  |  | S I | S I |  |  |  |  |  |  | S |  |  |  |  |  |  |  |  |  |  |  |  |  |  |  |  |  |  |
